# Detection of Cases of Noncompliance to Drug Treatment in Patient Forum Posts: Topic Model Approach

**DOI:** 10.2196/jmir.9222

**Published:** 2018-03-14

**Authors:** Redhouane Abdellaoui, Pierre Foulquié, Nathalie Texier, Carole Faviez, Anita Burgun, Stéphane Schück

**Affiliations:** ^1^ Unité de Mixte de Recherche 1138 Team 22 Institut National de la Santé et de la Recherche Médicale / Université Pierre et Marie Curie Paris France; ^2^ Kappa Santé Innovation (Kap Code) Paris France; ^3^ Medical Informatics Hôpital Européen Georges-Pompidou Assistance Publique-Hôpitaux de Paris Paris France

**Keywords:** medication adherence, compliance, infodemiology, social media, text mining, depression, psychosis, peer-to-peer support, virtual community

## Abstract

**Background:**

Medication nonadherence is a major impediment to the management of many health conditions. A better understanding of the factors underlying noncompliance to treatment may help health professionals to address it. Patients use peer-to-peer virtual communities and social media to share their experiences regarding their treatments and diseases. Using topic models makes it possible to model themes present in a collection of posts, thus to identify cases of noncompliance.

**Objective:**

The aim of this study was to detect messages describing patients’ noncompliant behaviors associated with a drug of interest. Thus, the objective was the clustering of posts featuring a homogeneous vocabulary related to nonadherent attitudes.

**Methods:**

We focused on escitalopram and aripiprazole used to treat depression and psychotic conditions, respectively. We implemented a probabilistic topic model to identify the topics that occurred in a corpus of messages mentioning these drugs, posted from 2004 to 2013 on three of the most popular French forums. Data were collected using a Web crawler designed by Kappa Santé as part of the Detec’t project to analyze social media for drug safety. Several topics were related to noncompliance to treatment.

**Results:**

Starting from a corpus of 3650 posts related to an antidepressant drug (escitalopram) and 2164 posts related to an antipsychotic drug (aripiprazole), the use of latent Dirichlet allocation allowed us to model several themes, including interruptions of treatment and changes in dosage.

The topic model approach detected cases of noncompliance behaviors with a recall of 98.5% (272/276) and a precision of 32.6% (272/844).

**Conclusions:**

Topic models enabled us to explore patients’ discussions on community websites and to identify posts related with noncompliant behaviors. After a manual review of the messages in the noncompliance topics, we found that noncompliance to treatment was present in 6.17% (276/4469) of the posts.

## Introduction

### Background

A report published by the World Health Organization (WHO) in 2003 highlighted that noncompliance (or nonadherence) to long-term treatment was a worldwide problem detrimental to the overall effectiveness of the health system [1]. Compliance is defined in this report as the degree of correspondence between a patient’s behavior (taking medications, following hygiene rules, and diet) and the recommendations made by a health care professional (HCP). Noncompliance with these recommendations has an impact on patients’ quality of life (QoL), outcomes, and health costs.

The WHO identified several causes of nonadherence to therapies, including the characteristics of the health system, the patient’s disease, and the course of treatment. For patients with depression, observance is linked to the frequency of administration of a drug and to concomitant therapy. For patients suffering from cancer, the fear of adverse effects (AEs) related to the treatment has negative impact on adherence. For diabetic patients, adherence may vary with age, sex, and the relationship with the physician. Several meta-analyses showed that current methods of improving medication adherence for chronic diseases were mostly complex and not very effective [2,3]. The Cochrane group concluded that (1) means to measure adherence more systematically and objectively and (2) innovations to assist patients to follow medication prescriptions for long-term medical disorders were major points to be considered in that field. Considering social media as platforms where patients can discuss about their treatments and share testimonies, they could be a new data source to measure adherence to treatment.

The use of social media allows large groups of people to create and share information, opinions, and experiences about health conditions and medications through discussions [4]. Social media provide pharmacovigilance experts with a relevant source of information [5]. The example of benfluorex [6] illustrated how social media could be valuable sources for experts. Methods to identify messages with adverse events mentions have been developed (eg, [7]).

Social media holds a lot of promise in improving communication and patient engagement [8]. Horvath et al [9] and Taggart et al [10] showed that information sharing and socializing with others were the criteria most often cited when HIV patients describe an ideal social network. Wang et al [11] modeled the discussions and interests of users of a forum for pregnant women using a topic model and showed that the women were sharing their experiences, fears, and concerns about medications. Stellefson et al [12] reviewed Web 2.0 interventions proposing a program of self-management to patients older than 50 years for their chronic disease. Patients highlighted the benefit of interacting with other patients. For example, sharing information through social networks enabled patients to communicate better with HCPs. Patients often use social media to discuss drug side effects and adherence to therapies. Mao et al [13] studied the messages from breast cancer patients treated by aromatase inhibitors. A total of 18.17% (4589/25,256) of the posts mentioned at least one adverse effect, and almost 12.8% (110/862) of the individuals mentioned discontinuing aromatase inhibitors. Chary et al [14] studied correlations between geographic distribution of prescription opioid misuse estimated from social media and the National Survey on Drug Usage and Health (NSDUH). They concluded that mentions of drug misuse on Twitter correlated strongly with the NSDUH estimates of opioid misuse.

Social media may even impact treatment adherence. In the study by Horvath et al [15], the results of a Web-based survey for HIV patients showed that 52.6% (164/312) of the participants were considered noncompliant. The meta-analysis published by Taggart et al [10] identified 2 studies on HIV populations that demonstrated a link between the use of social media and the improvement of compliance to treatment among users. Moreover, Mao et al [13] showed that breast cancer patients offer practical strategies to deal with drug side effects and provide support to each other. For example, 28.10% (7097/25,256) of the posts mentioned some method for addressing their aromatase inhibitor-related arthralgia, including exercising and pharmaceuticals, whether prescribed or over the counter.

Analysis of a huge number of narratives requires automated text mining techniques [5]. These techniques have been used to extract information from electronic health records. For example, Topaz et al [16] mined clinical narratives to identify heart failure patients who did not comply with their treatment. As for health records, detection of nonadherence behaviors in social media also requires text mining techniques.

Topic models could be used to discover hidden semantic structures in large sets of messages from social media. They could provide deeper exploration of nonadherence behaviors. This exploration is based on patient testimonies of their own decisions about drugs in real life.

### Objective

Our objective was to evaluate a topic model approach to identify messages describing noncompliant behaviors regarding medications. Topics correspond to clusters of words that represent the themes addressed by the patients. The distributions of these themes in a corpus of messages are expected to enable the targeted extraction of posts corresponding to noncompliance behaviors. We focused on two noncompliant behaviors: (1) dose change and (2) treatment cessation.

### Prior Work

Topic modeling is a text mining method designed for exploring the main topics that occur in a set of documents. With topic models, words that often occur together in text are grouped into different *topics*. On the basis of these topics, topic models provide a tool for unsupervised classification of massive collections of documents. Latent Dirichlet allocation (LDA) was developed by Blei et al as “...a generative probabilistic model for collections of discrete data such as text corpora...” [17].

Topic modeling algorithms have been used to analyze the thematic composition of text corpora extracted from social media in a variety of domains such as politics [18]. Several authors explored tweets content using LDA to identify health topics, including tobacco use [19], seasonal influenza and allergies [15], and childhood obesity [20]. Sullivan et al analyzed users’ comments from amazon to build a scoring system for food supplements [21].

Patient forums have been also explored using LDA. Yang et al [7] analyzed 1500 messages from patient forums to detect adverse drug reactions. The distributions of the themes obtained by applying the LDA model to this corpus made it possible to use similarity measurements for the annotated corpus compared with new messages. The authors proposed a message classifier based on these measurements. Noticeably, all the studies described above used messages in English.

With the objective of analyzing patients’ QoL in breast cancer, Tapi Nzali et al [22] investigated posts from Facebook groups and a public French breast cancer forum using LDA modeling. They analyzed messages in French.

Several algorithms may be applied to use topic models. The original version of LDA modeling proposed by Blei et al [17] has been widely used (eg, [7,19,20,22,23]). Paul and Dredze developed extensions of the LDA model [15,24,25]. To establish their *Ailment Topic Aspect Model* (ATAM), they added several components to associate a term with a theme (eg, a disease), or consider it as not relevant. Then, based on 144 million tweets, they estimated general themes and disease-specific themes such as influenza, cancer, and dental problems. The semantic coherence of the topics obtained by ATAM was better for 61% (11/18) of the estimated topics compared with the LDA [25].

In this study, we investigate the use of LDA to analyze the themes in patient posts and identify noncompliance cases. To the best of our knowledge, this is the first study aiming at identifying forum posts related to nonadherence behaviors.

## Methods

**Figure 1:**
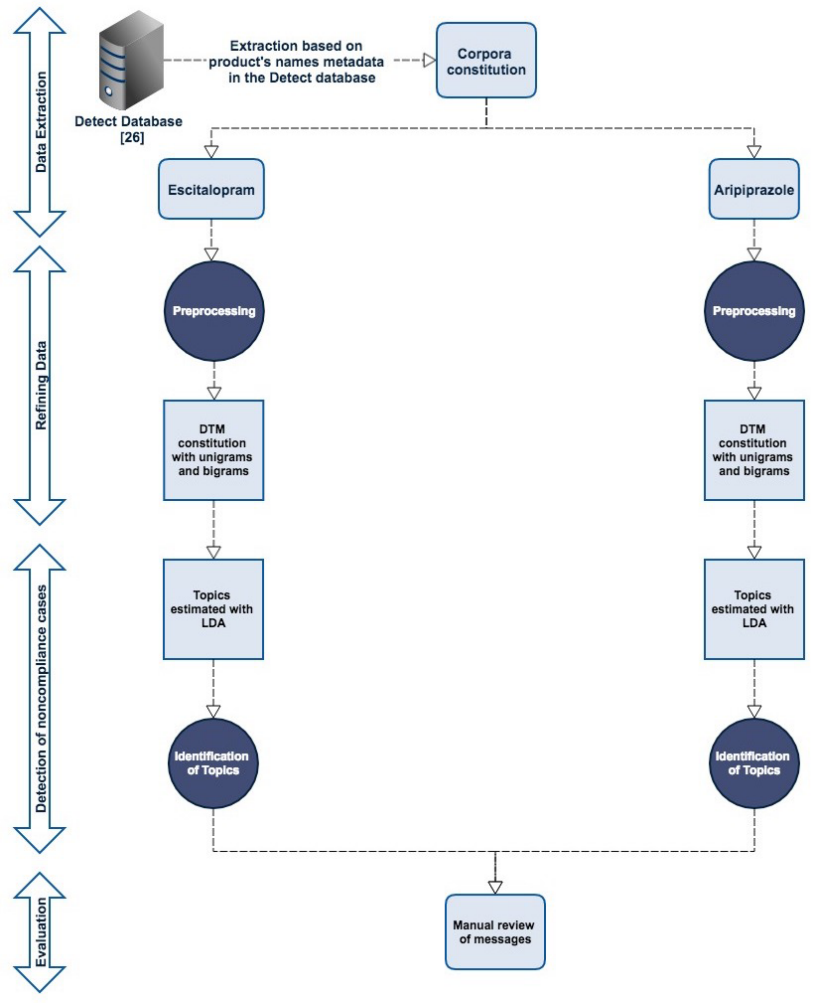
Summary diagram.

A summary of the approach presented in this study is provided in Figure 1.

### Materials

The data was extracted from the Detec’t database [26], a database developed by Kappa Santé [27] that collects messages from several French forums using a Web crawler. Detec’t extracts messages from forums based on a named entity recognition module using a drug lexicon made by Kappa Santé and a fuzzy matching algorithm. The lexicon was based on Racine Pharma and the Anatomical Therapeutic Chemical (ATC) classification system [28]. Racine Pharma is an extensive source of drug names that covers all medications available on the French market, including brand names and active ingredients. Racine Pharma entries are mapped to the ATC.

More precisely, we extracted two corpora from Detec’t: the first one corresponding to the messages related to escitalopram, an antidepressant drug, the other one related to aripiprazole, an antipsychotic drug. Rationale for choosing these drugs is that nonadherence cases are more likely to be found in chronic diseases and is a major concern in psychiatric disease management [29]. Moreover, these drugs belong to two different therapeutic classes: escitalopram is in a class of antidepressants called selective serotonin reuptake inhibitors; aripiprazole belongs to the so-called *atypical* second generation antipsychotics and acts as a partial dopamine agonist.

All messages extracted from Detec’t database in this study were posted from 2004 to 2013 on three of the most popular French forums (doctissimo, atoute, and santé médecine). The metadata accompanying each message that form the corpus were as follows: (1) an identifier, (2) the date of publication on the forum, and (3) the forum from which the message was extracted. Messages were extracted based on the respective brand names: Seroplex and Abilify of the drugs. Posts were selected based on the presence of the drug name in the message.

### Methods Used

#### Preliminary Data Processing

#### Preprocessing

The aim of the preprocessing step is the data cleaning to reduce noise and incoherence [30]. Preprocessing was done in six steps:

1. Considering that the R software (The R Project for Statistical Computing, Vienna) discriminates between lowercase and uppercase words, all messages were converted to lower case text.
2. The punctuation and stop words were removed.
3. We removed all instances of the drug name that was used to build the corpus (eg, seroplex). As it was present in each message, it was overrepresented and does not carry any further information.
4. Spaces were removed whenever needed to create *tokens*.
5. The stemming of words was carried out using Porter’s algorithm [31,32].
6. We decided to keep *unigrams* and *bigrams*. This made it possible to retain frequent contiguous sequences of two items, such as *effets secondaires* (AEs).

#### Standardization of Dosage Mentions

As variations in representing dosage in posts are possible (eg, milligram or mg), we replaced it by a standard expression in the messages: we identified dosage mentions (eg, *10 mg*) by searching each sequence of numbers followed by a dosage unit. Then, we replaced the dosage mention by a neutral string of characters *dosemilligrams*.

#### Model Estimation

##### Document-Term Matrix Weighting

The *document-term matrix* (DTM) describes the frequency of terms that occur in the collection of posts: rows correspond to posts (documents), and columns correspond to terms. If a term occurs in a particular post, then the matrix entry corresponding to that row and column is 1, if not it is 0. The sparsity corresponds to the frequency of zero-valued elements in the matrix.

A maximum sparsity threshold, above which the token was removed, was determined empirically. The total sparsity of the matrix was calculated for an interval of sparsity thresholds applied to the columns. These values ranged from 99.95% to 80% and decremented by 0.025%. We included tokens corresponding to a DTM sparsity of at least 97%. Then, to avoid overrepresentation of frequent tokens, we applied a weighting to our DTM based on the *term-frequency-inverse- document-frequency* approach [33]. One DTM was generated for each corpus (escitalopram and aripiprazole, respectively) and used as input of the topic modeling.

To remove the tokens that corresponded to spelling errors or abbreviations and consider only words frequently used by patients, we removed infrequent tokens based on DTM sparsity.

##### Latent Dirichlet Allocation Modeling

In this study, we decided to work with LDA algorithm. The model was described as follows by Blei and Lafferty [34]:

In LDA, the observed data are the words of each document and the hidden variables represent the latent topical structure, i.e., the topics themselves and how each document exhibits them...The interaction between the observed documents and hidden topic structure is manifest in the probabilistic generative process associated with LDA.

A document is a mixture of topics; that is, it corresponds to a probability distribution over all topics in the corpus. In other terms, when a patient writes a message, she or he decides to talk about a certain number of topics. When she or he talks about a topic in a message, she or he takes words with a certain probability from the set of terms that correspond to that topic. Assuming that model, each message contains several topics among all the identified topics, and the probability distribution shows how prominent the identified topics are in this message.

From a technical standpoint, rationale for choosing LDA was threefold:

- Compared with other types of topic modeling (latent semantic analysis, LSA; nonnegative matrix factorization, NMF; or singular value decomposition applied in the context of LSA), LDA methods are more suited in domains where data is in *semantic units*, such as words.
- LDA provides better interpretability of topics than other types of topic modeling (such as NMF).
- LDA also provides a better semantic coherence of estimated topics than LSA [35].

More precisely, we applied topic modeling with LDA algorithm developed by Blei et al [17,34]. The LDA model was estimated using the maximum a posteriori (MAP) algorithm described by Taddy [36,37]. MAP algorithm is a variant of expectation-maximization (EM) algorithm with a lower calculation cost and more stable results than the algorithms commonly used for estimates (Gibbs sampling, variational EM). At each iteration, instead of approximating the maximization of marginal likelihood, a combined estimate of the parameters is calculated by block-diagonalization of the Hessian matrix. This leads to an exact estimate of the distribution of topics, rather than an approximation. The number of topics was selected using the log Bayes factor [36]. Log Bayes factor is a ratio of likelihood used for model comparisons. By computing it against a one-topic model for several numbers of topics, it allows to select the most appropriate number. The output is twofold: (1) the probabilities of appearance associated with vocabulary terms in each of the *topics* and (2) the distribution thereof in the messages.

With the aim of optimizing interpretability and semantic coherence of topics, we considered a message significantly associated to a topic when at least 25% of the tokens it contained were associated to this topic. The 25% threshold was set empirically.

### Evaluation

The aim of the evaluation step was to assess the number of messages correctly identified by our approach. Manual evaluation was performed in two steps:

1. We reviewed manually all messages related to the topics of interest (*dosage variation* and *treatment interruption*) in the two corpora (Escitalopram and Aripiprazole). A message is considered correctly classified if it describes a noncompliant behavior corresponding to the recognized topic. The evaluation of our classification was measured by the ratio of correctly classified messages for each topic of interest. Two annotators (RA and PF) participated to the review. To measure interannotator agreement (IAA), the two annotators evaluated a random selection of 20% of posts from each set of messages identified by the noncompliance topics. The IAA was calculated using Cohen kappa coefficient [38].
2. To estimate sensitivity or recall of our method, we randomly extracted 20% of the messages related to topics other than noncompliance (345/1723 messages for aripiprazole and 650/3246 for escitalopram). We manually classified them in two categories: messages with noncompliance behaviors and without.

#### Software

Analyses were performed using the R software. For the preprocessing of the corpus, the packages *tm* [39], *SnowballC*, and *slam* were used. Topic models were estimated using the following packages: *topicmodels* [40] and *MAPTPX*.

## Results

### Datasets Characteristics

Table 1 shows the number of messages in each corpus.

The preliminary preprocessing of escitalopram corpus returned a DTM of 3650 messages and 155,883 tokens (*unigrams* and *bigrams*). Setting the sparsity threshold at 99.35% (3626.275/3650), we obtained a DTM of 3649 messages and 1497 tokens. One message was removed because the terms it contained were particularly misspelled.

The processing of the aripiprazole corpus yielded a DTM of 2164 messages and 81,371 tokens. On the basis of a sparsity threshold of 99.25% (2147.77/2164), we obtained a DTM of 2164 messages and 1062 terms.

The *tokens* that appeared least frequently in the corpora were removed (Table 2).

### Dosage Variations and Treatment Discontinuation

#### Model Estimation

**Figure 2:**
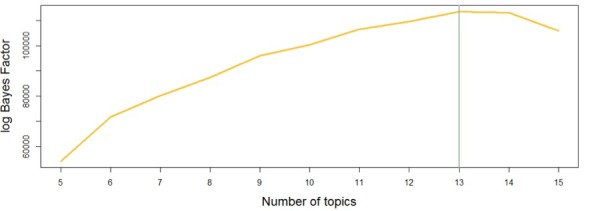
Number of topics selected for Escitalopram using the log Bayes factor.

The log Bayes factor topic selection method returned a total of 13 topics for the escitalopram corpus, as shown in Figure 2. The same approach led us to identify 11 topics for the aripiprazole corpus.

We obtained a total of 2691 messages evoking escitalopram and belonging to 13 topics. The 958 remaining messages were below the threshold regarding the association between terms and topics, which was set at 25%. The average number of topics per message was 1.22 and the median 1.

For the aripiprazole data, we obtained a total of 1778 messages mentioning the drug and distributed among 11 topics. The 396 remaining messages were below the threshold for association between message terms and topics. The average number of topics per message was 1.31 and the median 1.

#### Topics Interpretation

As a topic must be interpretable with the first terms obtained (ranked by their probability of appearance) [34], topics found were labeled manually based on the first 15 words.

**Table 1:** Corpora description.

Drug	Therapeutic class	Number of messages containing the drug name, n	Date of publication
Escitalopram	Antidepressant	3650	2004 to 2013
Aripiprazole	Antipsychotic	2164	2005 to 2013

**Table 2:** Description of the document-term matrix (DTM) dissemination thresholds.

Drug	Term frequency before processing, n	Sparsity before processing, n (%)	Sparsity threshold per token, n (%)	Term frequency after processing, n	Sparsity after processing, n (%)
Escitalopram	155,883	155,774 (99.93)	3626.275 (99.35)	1497	151,097 (96.93)
Aripiprazole	81,371	81,281 (99.89)	2147.77 (99.25)	1062	78,922 (96.99)

##### Escitalopram Topics

The list of topics and the distribution of messages in topics regarding escitalopram are displayed in Multimedia Appendix 1. This result is expressed as frequencies and proportions of messages (in relation to the 3649 escitalopram messages) associated with each theme. A message is associated with a topic if it contains at least 25% of terms for which the corresponding latent variability describes an association with the topic in question.

We noticed the emergence of a class containing messages describing user’s experiences with the drug in a general way (topic 7) and how it affects their condition (topic 6). Topics 3 and 8 related to the day-to-day feeling of patients and the activities they have. Topic 2 was linked to the drug prescription by HCPs and topic 5 to panic attacks and anxiety. Topics 9, 10, and 12 focused on messages about AEs experienced or feared by users, along with the drug’s effects overall. Topic 13 was related to the duration of the treatment.

Topics 1 was labeled as *general themes*. It describes themes associated with discussions between individuals and corresponds to poorly informative vocabulary. Such a collection of words provided no information of interest for our study. Nevertheless, it was widely used in messages, which explains the relatively high proportion of messages associated to this topic.

Messages about problems with treatment discontinuation and dosage variations were respectively included in topics 4 and 11. The intersection of the two noncompliance topics corresponded to 7 messages.

##### Aripiprazole Topics

The topics obtained by reproducing the modeling steps with the aripiprazole corpus are described in Multimedia Appendix 1.

Among the topics estimated for the aripiprazole corpus, we found the description of the patient's experience (topic 4) of his or her treatment. Three topics described the effects thereof (topics 3, 4, and 8), and one related to its duration (topic 9). Two topics focused on the patients’ relationships with HCPs (topic 5) and other individuals (topic 6). Topic 7 described treatment interruption. Dosage variations were described in topic 1. The intersection of the two noncompliance topics (7 and 1) corresponded to 6 messages. As for escitalopram, two topics were composed of noninformative words (general themes).

Multimedia Appendix 2 shows the identified topics in the two corpora.

### Evaluation of the Approach

IAA rate was measured on 20% (169/844) of the messages identified by the noncompliance topics using Cohen kappa coefficient. We obtained a kappa of 0.90 (152/169).

**Table 3:** Annotations of the escitalopram corpus.

Identified behavior	Number of messages, n	Number of correctly classified messages, n	Precision, %
Dosage variations	187	54	28.9
Treatment cessation	216	100	46.3

**Table 4:** Annotations of the aripiprazole corpus.

Identified behavior	Number of messages, n	Number of correctly classified messages, n	Precision, %
Dosage variations	176	56	31.8
Treatment cessation	265	62	23.4

We calculated the ratio of messages corresponding to a case of noncompliance associated with each topic of interest. The results are displayed in Tables 3 and 4. Globally, the precision score for noncompliance was 32.6% (272/844). We obtained the lowest score (23.4%, 62/265) for the aripiprazole discontinuation topic and the highest score (46.3%, 100/216) for escitalopram discontinuation.

The analysis of 20% (345/1723 for aripiprazole, 650/3246 for escitalopram) of the messages related to other topics than noncompliance revealed only four messages describing a nonadherence behavior and not detected by our approach. The four false negative messages were all about stopping aripiprazole. Regarding the different subsets, we obtained a 94% (62/66) recall score for the aripiprazole cessation topic and 100% for the other ones. Globally, the estimated recall score was 98.5% (272/276).

We present below a detailed analysis of the results for the aripiprazole corpus.

#### Dosage Variations

Topics modeling identified 176 messages as *dosage variations* messages. Manual review revealed that only 56 (31.8%, 56/176) messages contained a true noncompliance declaration (2.6% of the 2164 posts initially in the corpus).

Among the 120 remaining messages, 68 (56.7%, 68/120) were discussions between patients comparing their dosages for aripiprazole. A total of 13 messages (10.8%, 13/120) contained information regarding the dosages of other prescribed drugs in addition to aripiprazole. The most cited drugs were amisulpride and olanzapine. Eight posts (6.7%, 8/120) were questions about aripiprazole’s dosages, seven messages (5.8%, 7/120) evoked dosages modification, six posts (5.0%, 6/120) reported a dosage modification in agreement with the physician, and four messages (3.3%, 4/120) were advices.

Eight messages (6.7%, 8/120) did not contain dosage mentions but only variation words such as *increase* or *decrease*, usually accompanying dosage references.

The remaining six posts (5.0%, 6/120) mentioned variations that were planned or could occur in the future. For example:

[...] take 5 mg also for the moment the psy wants to increase the dose to 10 mg at the next appointment [...]

In all the noncompliance cases (56 cases), the patient decreased the dose because of adverse drug reactions (ADRs). The most frequent ADRs mentioned in these posts were insomnia, asthenia, and libido problems.

#### Treatment Cessation

We identified 62 messages corresponding to noncompliance behaviors out of the 265 posts related to the *treatment cessation* topic (23.4%, 62/265). In other terms, 2.86% (62/2164) of the 2164 posts in the corpus are messages from patients taking aripiprazole who decided to stop their treatment.

Among the 203 remaining posts, thirteen posts (6.4%, 13/203) corresponded to the interruption of aripiprazole but were not cases of noncompliance: either aripiprazole was stopped to start another treatment, or the treatment cessation was decided by the physician.

A total of 89 posts (43.9%, 89/203) were written by patients who were prescribed this treatment in the past.

In 55 messages (27.1%, 55/203), the patient mentioned that she or he was reluctant to continue the treatment, mainly (74.6%, 151/203) because of ADRs. The most cited symptoms were insomnia, tiredness, libido problems, and nausea.

In 23 (11.3%, 23/203) posts, the patient was given more than one drug, and the post described the interruption of one of the other drugs (eg, in fourteen cases it was olanzapine that was stopped).

Eighteen posts were assigned erroneously to the treatment cessation topic because they contained terms like *stop*, although not reporting discontinuation of aripiprazole. These included 11 messages (5.4%, 11/203), where interruption was not related to any health topic such as in “[...] I’m stopped, like frozen, [...],” five messages corresponding to cessation of alcohol, narcotics, or smoking (and not aripiprazole; 2.5%, 5/203), and two posts (<1%, 1/203) where the patient stopped her or his diet or other activities.

The five remaining messages (2.5%, 5/203) were requests for advices mentioning a possible treatment cessation.

## Discussion

### Principal Findings

Our study shows that topic models are useful to identify subsets of messages reporting noncompliance behaviors.

The topic models approach detected cases of noncompliance behaviors with averages recall and precision scores of 98.5% (272/276) and 32.6% (272/844), respectively. We concluded that the topic modeling presented in our study was a valuable sensitive method to detect noncompliance. However, it lacks specificity. We identified several situations leading to false positives: (1) two experiencers in the same message (eg, Peter takes 100 mg, whereas John takes 200 mg); (2) events in different time slots (eg, the patient reports that the doctor wants to increase or decrease the dose at the next appointment); and (3) the action concerns something else than the drug (eg, another medication and smoking cessation). Moreover, in several false positives, cessation or modification was prescribed by the physician.

### Clinical Significance

We focused on escitalopram and aripiprazole used to treat depression and psychotic conditions, respectively.

#### Escitalopram

Almost one million individuals (2% of the overall population) initiated an antidepressant in France in 2011 [41]. Patients’ adherence to antidepressant therapy must be evaluated. The reasons behind patient nonadherence to antidepressants include patient factors (eg, concerns about side effects and fears of addiction), as well as poor follow-up by the clinician and lack of sufficient patient education [42]. Better understanding of the patients’ concerns about these medications can be achieved by exploring the messages in social media. We retrieved 2691 messages about escitalopram, among which 154 (5.71%, 154/2691) were noncompliance messages (Table 3). AEs were the most commonly cited reason for discontinuation and dose reduction. The more common side effects for escitalopram included nausea, weakness, dizziness, sleeping disorders, and sexual problems.

#### Aripiprazole

In a recently published review [43], a positive attitude toward medication at baseline in combination with good psychosocial function was the best predictor of objectively measured mean adherence over a 12-month period in patients with schizophrenia. AEs such as patient-reported cognitive impairment resulting from antipsychotic medication were predictors of nonadherence. Common side effects of aripiprazole also include weight gain, nausea, vomiting, changes in appetite, dizziness, drowsiness, feeling tired, and insomnia, among others. In our corpus, patients reported that such AEs were reasons for stopping the treatment or changing the dose.

Almost 7% (6.86%, 122/1778) of the posts in the aripiprazole corpus corresponded to noncompliance behaviors. All decisions to change the dose by the patient corresponded to decreasing the dose because of AEs. This result suggests that text mining methods must extract ADR information along with noncompliance annotation.

We calculated the rate of messages describing an effective noncompliance behavior. These rates were measured on messages corresponding to topics identified on the aripiprazole corpus. This evaluation resulted in 31.8% (56/176) for dosage variations and 23.4% (62/265) for treatment discontinuation.

Using topic models seems to be insufficient for identifying noncompliance cases on social media without a manual review step. However, this lexical approach produced only four false negatives and enabled us to reduce the corpus by focusing on messages that had a high probability to contain descriptions of targeted noncompliance behaviors.

### Limitations

Our study focused on two drugs from two distinct classes. Both drugs are used to treat psychiatric disorders. A review currently including 50 clinical studies and 9476 participants taking antipsychotic drugs revealed an overall attrition from the included studies of 49% [44]. Consequently, our results regarding the noncompliance rate and the reasons for not being compliant cannot be extrapolated to other patient profiles. Further studies on other therapeutic classes must be conducted.

Manual review was required to distinguish between true and false positives in each dataset. The vocabulary used to describe dosage modifications or treatment interruptions in messages is commonly employed for characterizing other kinds of general variations or cessations (diet, smoking, etc). Topic models demonstrated their ability to identify potential noncompliance messages (average recall 98.5%, 272/276). Syntactic and semantic methods could be developed to recognize the experiencers, the temporal features, and the object concerned by the action in the sentences. Such methods could be applied to the datasets identified by the topic models to reduce the number of false positives and improve the precision score.

Another limitation of our work is the empirical determination of the thresholds used in our method. The thresholds concern the reduction in the size of the DTM and the significance of the association of messages to topics:

1. The choice of a sparsity threshold under 97% for DTM does not guarantee the best compromise between the calculation cost and the preservation of information for all the corpora we used.
2. The threshold for association between message words and topics, which was set at 25%, led to 23.23% (1354/5813) of messages not related to any topic.

Such empirical approach in the application of these methods is frequently reported in the literature; for example, Prier et al [19] set a suitable number of topics for their corpus by testing thresholds set every 50 topics.

### Comparison With Other Work

Our study, to our knowledge, is the first one aiming at analyzing noncompliance behaviors from social media messages.

Most of the studies [18,21,22,25] used topic models to automatically label sets of tweets. Only 2 studies [7,22] focused on medical themes and messages from Web forums. Both used the same LDA model. Tapi Nzali et al [22] used the same R package [40]. However, their study design was different: they evaluated the correspondence between identified topics and QoL questionnaires, whereas our study aimed at detecting nonadherence behaviors.

Yang et al reported higher precision rates in their study [7]. Nevertheless, the aim of their study was detection of ADRs, not noncompliance practices.

Our approach could benefit from a more sophisticated model. The Structural Topic Model, developed by Wang et al [11], enables the modeling of correlations between topics and transitions made within messages. The additional components would enable the identification of relations between noncompliant practices and information, such as ADRs. We could therefore determine potential causes of nonadherence to treatment for each kind of drug.

### Conclusions

Topic distributions in messages are a way to classify posts and detect noncompliance behaviors. The topic modeling approach achieved very high recall (98.5%, 272/276). Manual review of the messages in the noncompliance topics showed that almost 6.17% (276/4469) of the posts written by patients taking aripiprazole or escitalopram revealed noncompliance to treatment (half of them stopping their treatment). These findings indicate that social media mining may contribute to better understand noncompliance attitudes.

## Appendix

Multimedia Appendix 1 Exhaustive description of identified topics for each medication.

Multimedia Appendix 2 Topics found in each corpus ranked by the number of messages.

## Abbreviations

ADR: adverse drug reaction
AE: adverse effect
ATAM: Ailment Topic Aspect Model
ATC: Anatomical Therapeutic Chemical
DTM: document-term matrix
EM: expectation-maximization
HCP: health care professional
IAA: inter-annotator agreement
LDA: latent Dirichlet allocation
LSA: latent semantic analysis
MAP: maximum a posteriori
NMF: nonnegative matrix factorization
NSDUH: National Survey on Drug Usage and Health
QoL: quality of life
WHO: World Health Organization
